# Supplementary material for: Scrub typhus association with autoimmune biomarkers and clinical implications
Source: PLoS Negl Trop Dis. 2025 Jan 29;19(1):e0012766. doi: 10.1371/journal.pntd.0012766 (PMC11778775; doi:10.1371/journal.pntd.0012766)
Supplement: S1 Table — (DOCX) [file pntd.0012766.s001.docx]

S1 Table. Subgroup Analysis of Demographic and Immunological Characteristics of Healthy Controls and Scrub Typhus Patients Aged 40-59 Years

|  | **Controls** | | **Scrub Typhus** | **Total** | ***p*** |
| --- | --- | --- | --- | --- | --- |
|  | **(N = 10)** | | **(N = 28)** | **(N = 38)** |  |
| **Gender, n (%)** | |  |  |  | 0.468 |
| F | | 6 (60.0) | 22 (78.6) | 28 (73.7) |  |
| M | | 4 (40.0) | 6 (21.4) | 10 (26.3) |  |
| **Age** | | 50.6 ± 4.9 | 53.4 ± 5.1 | 52.7 ± 5.1 | 0.143 |
| **Anti-dsDNA IgM (U/mL)** | | 11.1 ± 16.5 | 12.2 ± 12.2 | 11.9 ± 13.2 | 0.821 |
| **ANA titer, n (%)** | |  | | | 0.037 |
| < 1:80 | | 8 (80.0) | 11 (39.3) | 19 (50.0) |  |
| 1:80 | | 1 (10.0) | 6 (21.4) | 7 (18.4) |  |
| 1:160 | | 1 (10.0) | 8 (28.6) | 9 (23.7) |  |
| ≧ 1:320 | | 0 (0.0) | 3 (10.7) | 3 (7.9) |  |

ANA, anti-nuclear antibody; Anti-dsDNA IgM, Anti-double-stranded DNA IgM. Data are presented as mean ± S.D. or number (percentage).
