# Supplementary material for: Scrub typhus association with autoimmune biomarkers and clinical implications
Source: PLoS Negl Trop Dis. 2025 Jan 29;19(1):e0012766. doi: 10.1371/journal.pntd.0012766 (PMC11778775; doi:10.1371/journal.pntd.0012766)
Supplement: S2 Table — (DOCX) [file pntd.0012766.s002.docx]

**S2 Table. Logistic Regression Analysis of Factors Associated with Low Serum Albumin Levels (≤ 3.5 g/dL) in Patients with Scrub Typhus**

|  | Univariate analysis | | | | Multivariate analysis | | | |
| --- | --- | --- | --- | --- | --- | --- | --- | --- |
|  | P Value | OR | Lower CI | Upper CI | P value | OR | Lower CI | Upper CI |
| **Sex (Male)** | 0.682 | 1.20 | 0.51 | 2.83 |  |  |  |  |
| **Age** | 0.027 | 1.04 | 1.01 | 1.08 | 0.061 | 1.04 | 1.00 | 1.08 |
| **Titer categorization (<1:80, ≧ 1:80 and <1:320, ≧ 1:320)** | 0.007 | 2.09 | 1.24 | 3.63 | 0.016 | 1.95 | 1.14 | 3.44 |

CI, Confidence Interval; OR, Odds Ratio
