# Supplementary material for: Scrub typhus association with autoimmune biomarkers and clinical implications
Source: PLoS Negl Trop Dis. 2025 Jan 29;19(1):e0012766. doi: 10.1371/journal.pntd.0012766 (PMC11778775; doi:10.1371/journal.pntd.0012766)
Supplement: S3 Table — (DOCX) [file pntd.0012766.s003.docx]

**S3 Table. Logistic Regression Analysis of Factors Associated with Low C4 Levels (≤ 40 mg/dL) in Patients with Scrub Typhus**

|  | Univariate analysis | | | | Multivariate analysis | | | |
| --- | --- | --- | --- | --- | --- | --- | --- | --- |
|  | P Value | OR | Lower CI | Upper CI | P value | OR | Lower CI | Upper CI |
| **Sex (Male)** | 0.055 | 3.15 | 1.06 | 11.66 | 0.106 | 2.73 | 0.87 | 10.53 |
| **Age** | 0.027 | 1.04 | 1.01 | 1.08 | 0.134 | 1.03 | 0.99 | 1.07 |
| **Titer categorization (<1:80, ≧ 1:80 and <1:320, ≧ 1:320)** | 0.012 | 2.26 | 1.23 | 4.42 | 0.030 | 2.06 | 1.10 | 4.09 |

CI, Confidence Interval; OR, Odds Ratio
