# Supplementary material for: Scrub typhus association with autoimmune biomarkers and clinical implications
Source: PLoS Negl Trop Dis. 2025 Jan 29;19(1):e0012766. doi: 10.1371/journal.pntd.0012766 (PMC11778775; doi:10.1371/journal.pntd.0012766)
Supplement: S4 Table — (DOCX) [file pntd.0012766.s004.docx]

**S4 Table. Logistic Regression Analysis of Factors Associated with high anti-dsDNA IgM Levels ( ≧ 15 IU/mL) in Patients with Scrub Typhus**

|  | Univariate analysis | | | | Multivariate analysis | | | |
| --- | --- | --- | --- | --- | --- | --- | --- | --- |
|  | P Value | OR | Lower CI | Upper CI | P value | OR | Lower CI | Upper CI |
| **Sex (Male)** | 0.430 | 0.72 | 1.31 | 1.59 |  |  |  |  |
| **Age** | 0.499 | 0.99 | 0.96 | 1.02 |  |  |  |  |
| **Titer categorization (<1:80, ≧ 1:80 and <1:320, ≧ 1:320)** | 0.001 | 2.30 | 1.41 | 3.91 | 0.001 | 2.30 | 1.41 | 3.91 |

CI, Confidence Interval; OR, Odds Ratio
