# Supplementary material for: Scrub typhus association with autoimmune biomarkers and clinical implications
Source: PLoS Negl Trop Dis. 2025 Jan 29;19(1):e0012766. doi: 10.1371/journal.pntd.0012766 (PMC11778775; doi:10.1371/journal.pntd.0012766)
Supplement: S5 Table — (DOCX) [file pntd.0012766.s005.docx]

**S5 Table. Logistic Regression Analysis of Factors Associated with high hs-CRP Levels ( ≧ 100 mg/L) in Patients with Scrub Typhus**

|  | Univariate analysis | | | | Multivariate analysis | | | |
| --- | --- | --- | --- | --- | --- | --- | --- | --- |
|  | P Value | OR | Lower CI | Upper CI | P value | OR | Lower CI | Upper CI |
| **Sex (Male)** | 0.190 | 1.79 | 0.76 | 4.35 | 0.346 | 1.54 | 0.63 | 3.83 |
| **Age** | 0.027 | 1.04 | 1.01 | 1.08 | 0.056 | 1.03 | 1.00 | 1.07 |
| **Titer categorization (<1:80, ≧ 1:80 and <1:320, ≧ 1:320)** | 0.200 | 1.39 | 0.84 | 2.33 | 0.385 | 1.26 | 0.75 | 2.14 |

CI, Confidence Interval; OR, Odds Ratio
