# Supplementary material for: Scrub typhus association with autoimmune biomarkers and clinical implications
Source: PLoS Negl Trop Dis. 2025 Jan 29;19(1):e0012766. doi: 10.1371/journal.pntd.0012766 (PMC11778775; doi:10.1371/journal.pntd.0012766)
Supplement: S6 Table — (DOCX) [file pntd.0012766.s006.docx]

**S6 Table. Proportion of Anti-dsDNA IgM Positivity (≥ 15 U/mL) According to ANA Titer Levels in Patients with Scrub Typhus**

|  | **< 1:80**  **(N = 40)** | **≧ 1:80, <1:320**  **(N = 47)** | **≧ 1:320**  **(N = 52)** | **Total (N = 139)** | *P* |
| --- | --- | --- | --- | --- | --- |
| Anti-dsDNA IgM ≧ 15 U/mL N,(%) | 6 (15.0) | 11 (23.4) | 24 (46.2) | 41 (29.5) | 0.003 |

Anti-dsDNA IgM, Anti-double-stranded DNA Immunoglobulin M
