# Supplementary material for: Scrub typhus association with autoimmune biomarkers and clinical implications
Source: PLoS Negl Trop Dis. 2025 Jan 29;19(1):e0012766. doi: 10.1371/journal.pntd.0012766 (PMC11778775; doi:10.1371/journal.pntd.0012766)
Supplement: Attachment — Submitted filename: Comments on Manuscript PNTD-D-24-00644.pdf [file pntd.0012766.s007.pdf]

# Comments for Manuscript PNTD-D-22-01154

## 1 Summary

**Manuscript title**: Scrub Typhus Association with Autoimmune Biomarkers and Clinical Implications

**Manuscript type**: Research Article

**Main research question/contribution**: Authors are interested in showing that antinuclear antibodies (ANA) titer can serve as a biomarker for distinct scrub typhus disease attributes or immunological responses. Authors indicated that elevated antinuclear antibodies (ANA) and anti-double stranded DNA (anti-dsDNA) IgM titers were observed in patients diagnosed with scrub typhus in comparison to a control group comprising healthy individuals.

**Overall impression**: While the authors do an acceptable job on explaining concepts regarding the study design, variables, and data processing, the explanation and implementation of the statistical analysis need more work.

**Reviewer decision**: In its present form, the manuscript does not seem suitable for publication. Due to the importance of autoimmune diseases and potential correlates, this analysis serves a basis for further studies and analysis. Therefore, I encourage the authors to resubmit a revised manuscript, by providing a point-by-point response on how the comments below were addressed.

## 2 Specific comments

### 2.1 Major issues

- Statistical Analysis:
  - Authors need to provide more explanations about the statistical analyses being implemented using MedCalc.
  - Indicate the type of statistical tests used (t-test vs anova vs chi-square; univariate vs multivariate; please be specific) and the significance level (the figure legends indicate 5% level; please include this in the main text as well) for the interpretation of the resulted p-values.
  - The categorical variable 'ANA titer' poses a concern within the analysis, and need to be re-evaluated. Several levels of this variable have less than 5 or even zero subjects. Using this variable as shown leads to unreliable or unstable estimates. Authors are encouraged to consider the combination of these sparse categories with other similar categories to ensure that each level has a sufficient number of observations. For instance, combine 1:320, 1:640, and 1:1280 as a category >1:160, if domain knowledge supports this modification.
  - For the analyses in Figures 1 to 4, exact p-values are needed.

- From Figures 3 and 4, it seems authors are performing multiple comparisons (>=7days vs earlier times; 2 comparisons per Figure). Provide assessment of multiple comparison correction to control for the increased risk of Type I errors.
- The hypothesis mentioned in the Discussion section could be moved to Analysis section, so it gives more meaning to the article and why you are doing the work.

## 2.2 Minor issues

- While it may be clear to the authors and readers within the same research area, the following notations/abbreviations ('anti-dsDNA IgM' and 'SLE') in the abstract, need to be explained in the same manner as 'antinuclear antibodies (ANAs)'.
- Indicate that anti-dsDNA is antibody to double-stranded deoxyribonucleic acid.
- Define TSA as type-specific antigen.
- Define Multilocus sequence typing (MLST).
- Figures 1 to 4 need to be reproduced in higher quality.
