# Supplementary material for: Scrub typhus association with autoimmune biomarkers and clinical implications
Source: PLoS Negl Trop Dis. 2025 Jan 29;19(1):e0012766. doi: 10.1371/journal.pntd.0012766 (PMC11778775; doi:10.1371/journal.pntd.0012766)
Supplement: Attachment — Submitted filename: Response to reviewers comments.docx [file pntd.0012766.s008.docx]

**Response to Reviewers’ Comments**

Dear Reviewers,

We would like to extend our deepest gratitude for your thorough review and valuable feedback on our manuscript. Your insightful comments and constructive suggestions have been instrumental in enhancing the quality and clarity of our study. We truly appreciate the time and effort you have dedicated to evaluating our work.

In response to your comments, we have meticulously revised and enhanced the manuscript to address the concerns and suggestions you have raised. We believe that these revisions have significantly strengthened our study, and we are sincerely grateful for your expert guidance throughout this process.

**Reviewer 2:**

**Method**

1. **Comment:** There is a significant difference in the age distribution between your control group and the scrub typhus group. You might need to address this in the methods section to enhance the credibility of the changes in autoantibodies after infection, which is the primary outcome of the article. This is because the proportion of ANA exceeding 1:80 is inherently higher in older individuals. Or you may consider the subgroup analyses based on age.

**Response**: Thank you for your insightful comment. We acknowledge the potential impact of age distribution on ANA titers. In response to your suggestion, we have made the following changes:

- We have added a detailed explanation in the Methods section to address the age distribution differences. Specifically, we have included a statement that highlights the need to account for age-related variations in ANA titers.

*“ To address the potential confounding effect of age distribution differences between the control group and the scrub typhus group on ANA titers, we included age as a key variable in our analysis. The age range of patients with scrub typhus was from 22 to 89 years, while the age range of the healthy controls was from 20 to 65 years. Recognizing that ANA positivity is inherently higher in older individuals, we implemented the following measures:*

- ***Age Stratification****: We stratified the study population into age groups to analyze the impact of age on ANA titers more precisely. Specifically, we conducted subgroup analyses focusing on patients aged 40-59 years. This age range was selected to ensure a sufficient sample size for statistical analysis while minimizing the age difference between groups.*
- ***Subgroup Analysis****: We compared the ANA titers within these age-defined subgroups. The demographic and immunological characteristics of healthy controls and patients with scrub typhus aged 40-59 years are presented in Supplemenatary Table 1. This analysis allowed us to evaluate the relationship between scrub typhus infection and ANA titers independently of age-related factors.*
- ***Statistical Adjustments****: In addition to subgroup analyses, we applied statistical adjustments to account for age as a covariate in our analysis of ANA titers. By including age as a covariate in our regression models, we aimed to control for its potential confounding effect and isolate the impact of scrub typhus infection on ANA titers.”*

Supplementary Table 1. Demographic and Immunological Characteristics of Healthy Controls and Patients with Scrub Typhus (Aged 40-59 years)

|  | **Controls** | | **Scrub Typhus** | **Total** | ***p*** |
| --- | --- | --- | --- | --- | --- |
|  | **(N = 10)** | | **(N = 28)** | **(N = 38)** |  |
| **Gender, n (%)** | |  |  |  | 0.468 |
| F | | 6 (60.0) | 22 (78.6) | 28 (73.7) |  |
| M | | 4 (40.0) | 6 (21.4) | 10 (26.3) |  |
| **Age** | | 50.6 ± 4.9 | 53.4 ± 5.1 | 52.7 ± 5.1 | 0.143 |
| **Anti-dsDNA IgM (U/mL)** | | 11.1 ± 16.5 | 12.2 ± 12.2 | 11.9 ± 13.2 | 0.821 |
| **ANA titer, n (%)** | |  | | | 0.037 |
| < 1:80 | | 8 (80.0) | 11 (39.3) | 19 (50.0) |  |
| 1:80 | | 1 (10.0) | 6 (21.4) | 7 (18.4) |  |
| 1:160 | | 1 (10.0) | 8 (28.6) | 9 (23.7) |  |
| ≧ 1:320 | | 0 (0.0) | 3 (10.7) | 3 (7.9) |  |

ANA, anti-nuclear antibody; Anti-dsDNA IgM, Anti-double-stranded DNA IgM. Data are presented as mean ± S.D. or number (percentage).

- To address the impact of age on ANA positivity, we conducted a subgroup analysis. The ages of patients with scrub typhus ranged from 22 to 89 years, while the ages of the healthy controls ranged from 20 to 65 years. To eliminate the effect of age on ANA positivity, we performed a subgroup analysis focusing on the age group of 40-59 years. The results are presented in the revised manuscript. The table below shows the demographic and immunological characteristics of healthy controls and patients with scrub typhus within the 40-59 age range. This analysis demonstrated that even within the same age range, patients with scrub typhus had significantly higher ANA titers compared to healthy controls, indicating that the observed differences are due to the infection itself rather than age-related factors. Also, We have added a detailed explanation regarding the selection criteria for the healthy control group in the Methods section to address this issue.

Please refer to the revised Methods section on page 5, line 124. The revised supplementary table can be found on page 1 under the name "Supplemental Data”.

2. **Comments:** Since the study examines temporal variation in antibody titers, specifying the follow-up duration and intervals for patient monitoring in methods would provide clearer insights into the longitudinal aspect of the study.

**Response:** Thank you for your valuable comment. We agree that specifying the follow-up duration and intervals for patient monitoring will provide clearer insights into the longitudinal aspect of our study. In response, we have added detailed information in the Methods section regarding the follow-up duration and the intervals used for antibody titer analysis.

*“* *In our study, antibody titer tests were conducted from the first day of symptom onset up to a maximum of 40 days. The follow-up periods were segmented into three distinct intervals: 1-3 days, 4-7 days, and beyond 7 days. This segmentation allowed us to analyze the variations in ANA and anti-double-stranded DNA IgM titers based on the temporal progression post-symptom onset.”*

Please refer to the revised Methods section on page 8, line 180.

3. **Comments:** May consider using multivariate regression analysis to control for confounding factors and more clearly determine the independent relationship between ANA titers and other clinical and laboratory parameters.

**Response:** Thank you for your valuable suggestion. In response to your comment, we have included a detailed explanation in the Methods section regarding the implementation of multivariate regression analysis to control for confounding factors. The multivariate regression analysis was conducted as follows:

*“**We performed logistic regression analyses to evaluate the independent relationship between ANA titers and various clinical and laboratory parameters. The analysis included adjustments for potential confounders such as age and sex.* *For the multivariate analysis, we included variables that had a p-value of up to 0.2 in the univariate analysis.* *The results of these analyses are summarized supplementary table 2-5.”*

The results of these analyses are summarized in the following supplementary tables:

Supplementary Table 2. Logistic Regression Analysis of Factors Associated with Low Serum Albumin Levels (≤ 3.5 g/dL) in Patients with Scrub Typhus

Supplementary Table 3. Logistic Regression Analysis of Factors Associated with Low C4 Levels (≤ 40 mg/dL) in Patients with Scrub Typhus

Supplementary Table 4. Logistic Regression Analysis of Factors Associated with High anti-dsDNA IgM Levels (≥ 15 IU/mL) in Patients with Scrub Typhus

Supplementary Table 5. Logistic Regression Analysis of Factors Associated with High hs-CRP Levels (≥ 100 mg/L) in Patients with Scrub Typhus

These details have been added to ensure clarity and accuracy in the interpretation of our results. Please refer to the revised Methods section on page 9, line 198 and Results section on page 14, line 285, for these updates. The supplementary tables can be found under "Supplemental Data."

**Results**

4. **Comments:** The difference of age group with the trend of ANA titer in Table 3 indicate the autoantibodies being affected by age. I suggest breaking down the proportions of several important ANA patterns, especially in cases where ANA > 1:80, to clarify whether there are patterns consistent with ds-DNA.

**Response:** Thank you for your insightful comment. We acknowledge the importance of analyzing ANA patterns to determine consistency with ds-DNA antibodies. However, in our study, we did not analyze specific ANA patterns; rather, we focused on the titer levels. Therefore, it is difficult to confirm whether the ANA patterns are consistent with ds-DNA antibodies.

To address your concern, we have conducted additional analyses to examine the proportion of ds-DNA antibody positivity according to the ANA titer levels. The results of this analysis are presented in the table below. This analysis provides a clearer understanding of the relationship between ANA titers and ds-DNA antibody positivity.

**Supplementary Table 6. Proportion of Anti-dsDNA IgM Positivity (≥ 15 U/mL) According to ANA Titer Levels in Patients with Scrub Typhus**

|  | **< 1:80**  **(N = 40)** | **≧ 1:80, <1:320**  **(N = 47)** | **≧ 1:320**  **(N = 52)** | **Total (N = 139)** | ***P*** |
| --- | --- | --- | --- | --- | --- |
| Anti-dsDNA IgM ≧ 15 U/mL N,(%) | 6 (15.0) | 11 (23.4) | 24 (46.2) | 41 (29.5) | 0.003 |

Anti-dsDNA IgM, Anti-double-stranded DNA Immunoglobulin M

This additional analysis highlights the distribution of ds-DNA antibody positivity across different ANA titer levels. We hope this addresses your concern and provides further clarity on the relationship between ANA titers and ds-DNA antibodies.

Please refer to the revised Results section on page 13, line 290 and revised supplementary materials for the detailed table.

**Conclusions**

5. **Comments:** As my opinion, ANA is an important screening tool and entry criterion for diagnosing SLE, with high sensitivity, low specificity. You may need focus on the potential pathogenesis why scrub typhus infection trigger early pathologic course of SLE due to you try to enhance the link between them.

**Response:** Thank you for your insightful comments. We appreciate your suggestion to explore the potential pathogenesis of how scrub typhus infection could trigger the early pathological course of systemic lupus erythematosus (SLE).

In our study, we observed elevated antinuclear antibodies (ANA) and anti-double stranded DNA (anti-dsDNA) IgM titers in patients diagnosed with scrub typhus compared to a control group of healthy individuals. These findings are consistent with the hypothesis that infections can act as environmental triggers for autoimmune responses. As detailed in our discussion, the progression of scrub typhus is marked by an increased intensity of immune responses, as evidenced by the temporal variation in antibody titers following symptom onset.

To address your comment specifically, while we did not analyze specific ANA patterns to directly correlate them with ds-DNA, our study did reveal significant changes in antibody titers over the course of the disease. We found that 21.5% of scrub typhus patients had ANA titers ≥ 1:320, and 37.4% showed positivity at titers ranging from 1:160 to 1:1280, which suggests that the elevated ANA titers observed in patients with scrub typhus cannot be solely attributed to aging.

Additionally, we observed that late-stage scrub typhus infection showed meaningful changes in anti-dsDNA IgM and C4 levels, which are markers relevant to SLE. This supports the notion that scrub typhus can induce an immune response that resembles the autoimmune activity seen in SLE. The observed clinical and immunological overlap between scrub typhus and SLE, such as fever, anemia, and thrombocytopenia, raises intriguing questions about the potential for infections to precipitate or exacerbate autoimmune responses in predisposed individuals.

Therefore, we have added the following section on the potential pathogenesis to the Discussion.

“*Our results suggest that scrub typhus infection could potentially trigger the early pathological course of SLE. This potential link may be explained by the release of sequestered antigens into the circulation due to cell and tissue damage during the vasculitic phase of scrub typhus. Such antigen release could stimulate an autoimmune response, resulting in elevated levels of ANA and anti-dsDNA antibodies. Furthermore, the clinical manifestations of scrub typhus, such as fever, anemia, and thrombocytopenia, mimic those seen in SLE, supporting the hypothesis that scrub typhus may trigger autoimmune processes in genetically predisposed individuals.”*

Please refer to the Discussion section on page 16, line 332.

**Reviewer 3:**

1. **Comments:** objective of the study is not articulated .indication of doing ANA titre and anti DS DNA in a diagnosed case of scrub typhus is not clear.

**Response:** Thank you for your valuable feedback. We appreciate your comments and would like to clarify the objectives of our study and the rationale for measuring ANA titer and anti-dsDNA antibodies in patients diagnosed with scrub typhus.

The primary objective of our study was to investigate whether scrub typhus, due to its vasculitic pathogenesis, could potentially trigger autoimmune responses. Specifically, we examined ANA, a representative autoantibody associated with autoimmune diseases. Since elevated ANA is a central screening marker for lupus, we also measured anti-dsDNA antibodies, which are highly specific for lupus, to explore the potential development of SLE among autoimmune diseases.

The rationale for measuring these autoantibodies in scrub typhus patients is based on the hypothesis that infections can act as environmental triggers for autoimmune responses. By evaluating the levels of these autoantibodies, we aimed to explore the potential link between scrub typhus infection and the early pathological processes of autoimmune diseases. Elevated levels of autoantibodies in scrub typhus patients could suggest that the infection induces an autoimmune response, providing insights into the complex interplay between infectious diseases and autoimmunity.

We have revised the Introduction sections to better articulate the study's objectives and the reasons for including the autoantibodies measurements. Please refer to the revised manuscript for these updates.

*“The primary objective of our study was to investigate whether scrub typhus, due to its vasculitic pathogenesis, could potentially trigger autoimmune responses. Specifically, we aimed to elucidate the development of autoimmunity by examining the expression of ANAs and associated autoantibodies, such as anti-dsDNA, across the different stages of scrub typhus infection and to assess their clinical significance. The rationale for measuring these autoantibodies in scrub typhus patients is based on the hypothesis that infections can act as environmental triggers for autoimmune responses. By evaluating the levels of these autoantibodies, we aimed to explore the potential link between scrub typhus infection and the early pathological processes of autoimmune diseases.”*

Please refer to the revised Introduction section on page 5, line 104.

**Specific Comments**

**2.1 Major Issues**

**∎ Statistical Analysis:**

- **Comments:** Authors need to provide more explanations about the statistical analyses being implemented using MedCalc.

**Response:** Thank you for your insightful comments. We apologize for the oversight in mentioning the software used for statistical analyses. The analyses were actually performed using IBM SPSS Statistics for Windows, version 25.0 (IBM Corp., Armonk, NY, USA), not MedCalc. This has been corrected in the revised manuscript page 9 line 194.

- **Comments:** Indicate the type of statistical tests used (t-test vs anova vs chi-square; univariate vs multivariate; please be specific) and the significance level (the figure legends indicate 5% level; please include this in the main text as well) for the interpretation of the resulted p-values.

**Response:** Regarding the statistical analyses, we have provided more detailed explanations in the Methods section. Specifically, we have included the following:

- Type of Statistical Tests Used: We have specified the types of statistical tests employed, including t-tests for comparing means between two groups, ANOVA for comparing means among three groups, and chi-square tests for examining the relationship between categorical variables. Additionally, we have detailed the use of univariate and multivariate logistic regression analyses. For the multivariate analysis, we included variables that had a p-value of up to 0.2 in the univariate analysis.

- Significance Level: The significance level for interpreting the p-values has been explicitly mentioned as 5%, consistent with the figure legends.

These details have been added to revised Method section page 9 line 194.

- **Comments:** The categorical variable ‘ANA titer’ poses a concern within the analysis and need to be re-evaluated. Several levels of this variable have less than 5 or even zero subjects.

Using this variable as shown leads to unreliable or unstable estimates. Authors are encouraged to consider the combination of these sparse categories with other similar categories to ensure that each level has a sufficient number of observations. For instance, combine 1:320, 1:640, and 1:1280 as a category >1:160, if domain knowledge supports this modification.

**Response:** Thank you for your valuable feedback and insightful suggestion. We appreciate your observation regarding the categorical variable ‘ANA titer’ and acknowledge the issue of sparse data in several levels of this variable. In response to your comment, we have re-evaluated the ‘ANA titer’ variable. While we understand the importance of combining sparse categories to ensure reliable and stable estimates, we also believe it is important to present detailed data in Table 1 to show the specific distribution of high-titer ANA levels. This detailed presentation highlights the higher frequency of these high-titer levels in scrub typhus patients compared to the control group. Therefore, to balance both needs, we have kept the detailed categories (1:320, 1:640, 1:1280) in Table 1 to provide a comprehensive view of the data.

Additionally, we acknowledge the validity of your statistical concern. Therefore, in the aging subgroup analysis (Supplementary Table 1), we have combined the categories >1:320 into a single category. This adjustment ensures that each level has a sufficient number of observations, thereby enhancing the robustness of the subgroup analysis.

We have revised the manuscript accordingly to reflect these changes. The updated analysis and the detailed presentation in Supplementary Table 1 together enhance the clarity and robustness of our results.

Please refer to the supplementary materials for the updated analysis and results. Thank you again for your constructive feedback.

- **Comments:** For the analyses in Figures 1 to 4, exact p-values are needed.

**Response:** Thank you for your valuable feedback. We appreciate your suggestion to include exact p-values for the analyses presented in Figures 1 to 4.

In response to your comment, we have revised the manuscript to include the exact p-values for all relevant analyses. The figures and their corresponding captions have been updated accordingly to reflect these changes. We believe that providing exact p-values enhances the transparency and precision of our statistical reporting.

Please refer to the revised manuscript for the updated figures and detailed *p*-values. Thank you again for your constructive feedback.

- **Comments:** From Figures 3 and 4, it seems authors are performing multiple comparisons (>=7days vs earlier times; 2 comparisons per Figure). Provide assessment of multiple comparison correction to control for the increased risk of Type I errors.

**Response:** Thank you for your insightful comment. We acknowledge the importance of controlling for the increased risk of Type I errors due to multiple comparisons. Initially, we conducted the Kruskal-Wallis test to compare multiple groups and determine if there were overall differences among them. Upon finding significant differences with the Kruskal-Wallis test, we conducted pairwise comparisons using the Mann-Whitney test. To account for the increased risk of Type I errors due to these multiple comparisons, we applied the Bonferroni correction method to adjust the p-values. The adjusted p-values have been calculated and included in the revised manuscript. Additionally, we have updated Figures 3 and 4 to reflect these values.

We have also included a detailed description of the multiple comparison correction method in the Methods section. These adjustments help ensure the robustness and reliability of our statistical analyses.

Please refer to the revised manuscript for the updated figures and detailed explanations of the multiple comparison corrections on page 9 line 200. Thank you again for your valuable feedback.

- **Comments:** The hypothesis mentioned in the Discussion section could be moved to Analysis section, so it gives more meaning to the article and why you are doing the work.

**Response**: Thank you for your valuable feedback. We appreciate your suggestion to move the hypothesis to the Analysis section to enhance the clarity and purpose of our study. In response to your comment, we have revised the manuscript accordingly.

Another reviewer, Reviewer 3, made a similar suggestion to clarify the hypothesis and objectives of our study. Therefore, we have modified the Discussion section and added the hypothesis and research objectives to the Introduction part. This adjustment aims to provide a clearer context for our work and better explain the rationale behind our investigation.

Please refer to the revised Introduction section on page 5, line 104 for the updated sections. Thank you again for your insightful comments.

**2.2 Minor Issues**

- **Comments:** While it may be clear to the authors and readers within the same research area, the following notations/abbreviations (‘anti-dsDNA IgM’ and ‘SLE’) in the abstract, need to be explained in the same manner as ‘antinuclear antibodies (ANAs)’.

**Response:** Thank you for your valuable feedback. We appreciate your observation regarding the clarity of abbreviations in the abstract.

In response to your suggestion, we have revised the abstract to include explanations for the abbreviations ‘anti-dsDNA IgM’ (anti-double stranded DNA Immunoglobulin M) and ‘SLE’ (Systemic Lupus Erythematosus), similar to how ‘antinuclear antibodies (ANAs)’ was introduced. This ensures that all readers, regardless of their familiarity with the field, can clearly understand the terminology used in our study.

Please refer to the revised manuscript for the updated abstract. Thank you again for your insightful comments.

- **Comments:** Indicate that anti-dsDNA is antibody to double-stranded deoxyribonucleic acid.

**Response:** Thank you for your valuable feedback. We appreciate your observation regarding the need to clarify the abbreviation ‘anti-dsDNA’. In response to your suggestion, we have revised the abstract and main text to indicate that ‘anti-dsDNA’ refers to antibodies to double-stranded deoxyribonucleic acid.

Please refer to the revised manuscript for the updated abstract and main text. Thank you again for your insightful comments.

- **Comments:** Define TSA as type-specific antigen. Define Multilocus sequence typing (MLST).

**Response:** Thank you for your valuable feedback. We appreciate your observation regarding the need to clarify the abbreviations ‘TSA’ and ‘MLST’. In response to your suggestion, we have revised the manuscript to define ‘TSA’ as type-specific antigen, which refers to antigens that are specific to a particular strain or type of microorganism, and ‘MLST’ as multilocus sequence typing, a method of characterizing isolates of microbial species using the sequences of internal fragments of multiple (usually seven) housekeeping genes.

Please refer to the revised manuscript for the updated definitions (page 6 line 123 and page 8 line 170). Thank you again for your insightful comments.

- **Comments:** Figures 1 to 4 need to be reproduced in higher quality.

**Response:** Thank you for your valuable feedback. We appreciate your observation regarding the quality of Figures 1 to 4. In response to your suggestion, we have reproduced these figures in higher quality, specifically at 300 – 600 dpi, to ensure that all details are clearly visible and the presentation is enhanced. The revised figures are now included in the manuscript.

**Closing comments**

We would like to express our sincere gratitude for your valuable feedback and insightful comments. Your suggestions have greatly contributed to the enhancement of our manuscript. We appreciate the time and effort you have dedicated to reviewing our work and helping us improve the quality and clarity of our study.

Thank you for your thorough review and constructive criticism. We believe that the revisions we have made in response to your comments have significantly strengthened our manuscript. We look forward to your continued feedback and support.
